# Descriptive study of interprofessional collaboration between physicians and osteopaths for the pediatric population in Quebec, Canada

**DOI:** 10.1186/s12913-017-2717-y

**Published:** 2017-11-14

**Authors:** Chantal Morin, Johanne Desrosiers, Isabelle Gaboury

**Affiliations:** 1 Department of Family Medicine and Emergency Medicine and School of Rehabilitation, Faculty of Medicine and Health Sciences, Université de Sherbrooke, Sherbrooke, Quebec, Canada; 2 School of Rehabilitation, Faculty of Medicine and Health Sciences, Université de Sherbrooke, Sherbrooke, Quebec, Canada

**Keywords:** Collaboration, Complementary medicine, Family medicine, Osteopathy, Pediatrics, Primary care, Referral, Survey

## Abstract

**Background:**

Osteopathy is an increasingly popular healthcare approach that uses a wide variety of therapeutic manual techniques to address pain and somatic dysfunction. In Quebec, Canada, osteopathy is the complementary medicine most often recommended by family physicians. However, factors fostering the development of interprofessional collaboration (IPC) between physicians and osteopaths are unknown. This study aimed to describe the current situation in terms of IPC among practitioners working with pediatric patients.

**Methods:**

A self-administered questionnaire was sent to osteopaths, family physicians, and pediatricians involved with pediatric patients in the province of Quebec. The postal questionnaire captured general knowledge about osteopathy and its practice parameters and role, sources of information, communication aspects including having a professional relationship and referrals, and influence of the upcoming government regulation. Quantitative data from the questionnaires were analyzed using descriptive statistics. Logistic regression model for factors associated with osteopathic referrals and multiple linear regression analyses for the number of correct answers about general osteopathic practice parameters were performed.

**Results:**

A total of 274 physicians (155 family physicians (response rate 13%) and 119 pediatricians (17%)) and 297 osteopaths (42%) completed the survey. According to physicians, osteopathy was most appropriate for musculoskeletal pain (241; 91%) and plagiocephaly (235; 88%). Osteopathic referral was positively associated with having a professional relationship (odds ratio [OR] 4.10 (95% confidence interval [CI] 2.12; 7.95), *p* < 0.001), personal consultation (OR 2.58 (95% CI 1.35; 4.93), *p* = 0.004), community-based practice (OR 1.89 (95% CI 1.03; 3.47), *p* = 0.040), and belief in the active role of osteopathy for pediatric conditions (OR 1.22 (95% CI 1.01; 1.47), *p* = 0.042). The majority of physicians (72%) and osteopaths (62%) considered the upcoming government regulation of osteopathy a positive factor for collaboration.

**Conclusion:**

Some collaboration already exists among these practitioners, including mutual referrals, but optimizing this collaboration still poses some challenges.

## Background

Osteopathy is a complementary and alternative medicine (CAM) that is growing in popularity in many countries including in Canada and especially in the province of Quebec [1, 2] where it is the CAM most often recommended by family physicians [3]. Osteopaths use a wide variety of therapeutic manual techniques to address pain and somatic dysfunction in order to restore the person’s natural state of physical well-being. Osteopathic manipulative treatments can facilitate the body’s normal self-regulation and self-healing mechanisms by addressing areas of tissue strain, stress, restriction, or dysfunction that may impede normal function [4]. In Quebec, osteopathic consultations for the pediatric population are frequent [5].The most common reasons include cranial deformities, torticollis, postnatal preventive healthcare, otolaryngology concerns, gastroesophageal reflux, motor or cognitive development concerns, musculoskeletal, respiratory and digestive problems, headaches, and sleep disturbances [2]. Such functional disorders are known to pose a significant challenge to conventional medicine [6–8] and frequently require an interprofessional approach [9, 10].

Interprofessional collaboration (IPC) in primary care may be defined as a set of relationships and interactions that allows professionals to share their knowledge, expertise and experience to concomitantly address complex client needs [11]. Many interactional (interpersonal relationships including willingness to collaborate, trust, communication and mutual respect), organizational and systemic factors (social, cultural, professional and education systems) are known to influence IPC [12]. In the context of IPC between conventional medicine and CAM, differences in work culture, paradigms, knowledge and language are factors preventing interactions and limiting collaborations [13]. Systemic factors such as liability concerns [14] and power disparities between physicians and CAM practitioners [13, 15, 16] also influence the collaboration process. There is no legislation yet controlling the training and the title, or regulating the practice of non-physician osteopaths in Quebec. In order to protect the public, the Office des professions du Québec (provincial regulatory body for all health care professions) is currently working towards a legislative framework to regulate the practice of osteopathy [17]. To date, no studies have been published about IPC between physicians and osteopaths for pediatric patients. The absence of studies about the factors enabling the development of IPC between physicians and osteopaths for pediatric and other patients, coupled with the increase in osteopathy consultations by parents, points to the need to study this working arrangement, especially given the pending governmental legislation of the osteopathic profession in Quebec.

This study aimed to describe the current general situation in the province of Quebec, Canada, in terms of IPC between physicians and non-physician osteopaths working with pediatric clients. More specifically, three descriptive aspects were examined: 1) physicians’ knowledge about osteopathic practice parameters and role; 2) communication including referrals and professional relationships; and 3) influence of the pending government regulation of osteopathy. In addition, factors associated with osteopathic referrals by physicians and factors linked to physicians’ knowledge of key osteopathic practice parameters were explored from descriptive data.

## Methods

### Study design, participants and recruitment procedure

This survey, conducted between September and November 2014, was the first (quantitative) phase of a larger sequential mixed method study aimed at improving understanding of IPC between physicians and non-physician osteopaths working with pediatric patients. Postal questionnaire were sent to all family physicians with a pediatric population and pediatricians without a subspecialty in Quebec, according to Scott’s MD Select 2013 directory as well as all members of Ostéopathie Québec (largest professional association in the province). A total of 2802 questionnaires were initially mailed to family physicians (*N* = 1327), pediatricians (*N* = 738) and osteopaths (*N* = 737). Assuming a 20% response rate for physicians/pediatricians and 40% for osteopaths, it is estimated that errors would be limited to 5% and 6% respectively, 19 times out of 20.

Efforts to maximize participation in the study included personalized mailing of questionnaires. The survey was also promoted on the Ostéopathie Québec website, and the first author attended various medical and osteopathic events in fall 2014. A reminder postcard was sent two weeks after the initial mailing and a second questionnaire two weeks later. Study aims were clearly described at the beginning of the questionnaire. Completing and returning the questionnaire anonymously indicate informed consent from participants. The study was approved by the Centre hospitalier universitaire de Sherbrooke ethics committee for health research on humans (#14–115).

### Instrument (survey questionnaire)

The physician and osteopath versions of the survey questionnaire were developed using a three-stage iterative process comprising developmental stage, question testing stage, and pilot stage [18].

The initial version of the questionnaire was based on a literature review of existing questionnaires regarding IPC between conventional medicine and CAM practitioners. It was divided into three categories according to the study’s conceptual model, the Chiropractor-physician model of collaborative practice of Mior et al., (2010): practice parameters, communication, and care delivery parameters including regulation [14]. We considered existing questionnaires on physicians’ [19] or other health professionals’ [20] knowledge of osteopathy, continuing education and sources of information about osteopathy [20] or CAM for the pediatric population [21], experiences of collaboration with CAM practitioners [21], referrals [19, 20, 22], communication and professional relationships [20], and sociodemographic variables related to collaboration [19–22]. This first version was pretested with two physicians, one pediatrician, three osteopaths, and an expert on IPC and questionnaire development. The modified version was piloted (procedure and duration) in individual face-to-face cognitive interviews with two physicians, two pediatricians and four osteopaths. It took 8 to 10 minutes to complete the survey. The final version of the questionnaires covered: a) knowledge about osteopathic practice parameters including 10 questions regarding general aspects, 10 questions concerning belief in the active role of osteopathy for specific pediatric conditions, and one question regarding sources of information about osteopathy (physician version only); b) communication aspects including interpersonal relationships, referrals, and communication methods (7 questions); c) influence of regulation of osteopathy on IPC (1 question); and d) sociodemographic data: gender, years of experience, type of practice, discipline, personal consultation of an osteopath (physician version), and presence of a physician in the working environment (osteopath version). Qualitative comments could be added by participants at the end of the questionnaire.

### Data analysis

The number of correct answers out of 10 was computed for the questions on the general aspects of practice parameters. Similarly, the total number of positive answers for the 10 questions on belief in the role of osteopathy for specific pediatric conditions was calculated. Means and standard deviations were generated. Other quantitative data from the questionnaires regarding communication, influence of regulation, and sociodemographic information were analyzed using descriptive statistics (frequencies and percentages).

Chi-squared or t tests (depending on the type of dependent variable) were first used to identify statistically significant variables associated with osteopathic referrals by physicians (yes or no) and factors associated with more correct answers out of 10 about general osteopathic practice parameters. Independent variables that were significantly (*p* < 0.05) associated were introduced in a stepwise backward logistic regression model for factors associated with osteopathic referrals or in multiple linear regression analyses for the number of correct answers about general osteopathic practice parameters. Normality of the knowledge variable was verified visually with a histogram and a residual analysis was conducted to verify basic assumptions for both regression models. All analyses were performed using SPSS 17 (Chicago, IL).

## Results

**Fig. 1:**
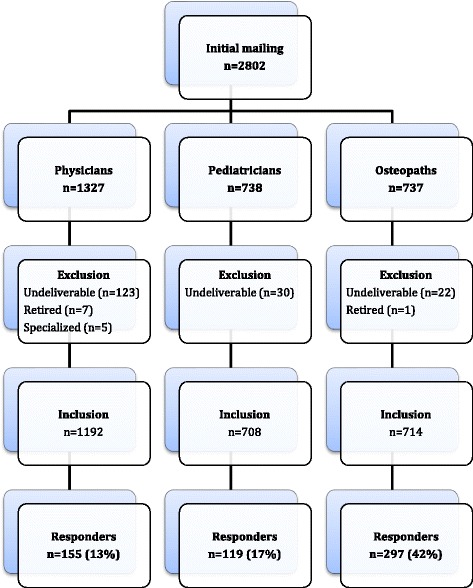
Response flowchart

After excluding undeliverable questionnaires, retired physicians and osteopaths and specialized family physicians (see Fig. 1), a total of 274 physicians (155 family physicians out of 1192 (response rate 13%) and 119 pediatricians out of 708 (response rate 17%)) and 297 osteopaths out of 714 (response rate 42%) completed the survey. All surveys were considered for analysis. The respondents’ characteristics are summarized in Table 1. The majority were women, 122 physicians (45%) had consulted an osteopath for themselves, and 38 osteopaths (13%) had a physician working in the same clinic.

**Table 1:** Characteristics of participants

Characteristics	Physicians (*n* = 274)	Osteopaths (*n* = 297)
	Freq. (%; 95% CI)^a^	Freq. (%; 95% CI)
Gender (female)	202 (73.7; 68.0, 78.8)	236 (79.5; 74.3, 83.8)
Working experience (yrs):		
*0–4*	22 (8.1; 5.3, 12.2)	55 (18.5; 14.4, 23.5)
*5–9*	34 (12.5; 8.9,17.2)	97 (32.7; 27.4, 38.4)
*10–14*	34 (12.5; 8.9, 17.2)	64 (21.5; 17.1, 26.8)
*15–20*	45 (16.5; 12.4, 21.6)	43 (14.5; 10.8, 19.1)
*+21*	137 (50.4; 44.3, 56.5)	38 (12.8; 9.3, 17.3)
Type of practice^b^:		
*Group*	75 (27.7; 22.5, 33.5)	159 (53.5; 47.7, 59.3)
*Solo*	25 (9.2; 6.2, 13.5)	159 (53.5; 47.7, 59.3)
*Hospital*	155 (57.2; 51.1, 63.1)	n/a
*Community practice*	132 (48.7; 42.6, 54.8)	n/a
*Rehabilitation centre*	9 (3.3; 1.6, 6.4)	n/a
*Other*	13 (4.8; 2.7, 8.3)	11 (3.7; 2.0, 6.7)

*n/a* not applicable

^a^Percentages reflect missing data (2 or 3 respondents)

^b^Respondents could check more than one answer

### Physicians’ knowledge about osteopathic practice parameters

Physicians correctly answered 7.2 (SD 1.8) questions out of 10 on average concerning general knowledge about the practice of osteopathy (Table 2). Level of osteopathic education and regulation status of the profession were the questions with the lowest percentage of correct answers. However, almost all physicians agreed that being a physical therapist is not a prerequisite for being an osteopath in Quebec and that osteopaths evaluate and mobilize all body tissues, not just vertebrae. There were 6.8 (SD 1.91) positive answers for belief in the role of osteopathy for specific pediatric conditions (Table 2). A high percentage of physicians think that osteopathy has a role in addressing musculoskeletal pain as well as torticollis and plagiocephaly. When asked what their sources of information about osteopathy are (multiple answers were allowed), more than half of the physicians mentioned asking information directly to an osteopath (52%), followed by patients (51%), and to other health professionals (21%). Information was also obtained by personal searches (17%), from scientific articles (10%), and during continuing education sessions (8%).

**Table 2:** Physicians’ general knowledge about osteopathic practice parameters and belief in the active role of osteopathy for specific pediatric conditions (*n* = 274)

Currently in Quebec, osteopaths …	Frequency of correct answers (%)^a^
Do not have a protected title	122 (46.2)
Have more hours of training than a college degree	202 (76.5)
Have more hours of training than a bachelor’s degree	147 (55.7)
Have training equivalent to a professional master’s degree	90 (34.1)
Should have WHO osteopathic educational standards	243 (92.0)
Do not evaluate and mobilize only vertebrae	267 (99.6)
Are not always physical therapists	264 (98.1)
Evaluate and mobilize all body tissues	255 (95.1)
Have extensive knowledge of anatomy, physiology and pathology	215 (81.1)
Work only with their hands	135 (50.9)
Do you agree that osteopathic intervention …	Frequency of positive answers (%)^a^
Is appropriate for musculoskeletal pain	241 (91.3)
Is appropriate for torticollis or positional plagiocephaly	235 (88.0)
Is not appropriate to evaluate recent, acute, or disabling abdominal pain	225 (85.9)
Is not appropriate to evaluate severe regurgitation with weight loss	221 (83.7)
Is not appropriate to relieve pain caused by otitis	206 (78.6)
Does not speed up the fracture healing process	183 (69.6)
Is appropriate for colic	138 (52.1)
Is appropriate for functional disorders (headache, foot alignment, etc.)	137 (51.9)
Is appropriate for general preventive healthcare	122 (46.4)
Is appropriate for postnatal preventive healthcare	114 (43.0)

*WHO* World Health Organization

^a^Percentages reflect missing data (5 to 11 respondents)

### Communication

Table 3 outlines communication aspects for each type of practitioner. More than one third of respondents had a professional relationship with the other practitioner. Nearly half the physicians referred pediatric patients to osteopaths at least once a month. The large majority of respondents (81% of physicians and 85% of osteopaths) said that communication was required for common patients and that their preferred communication methods were letters or verbal communication through the patient.

**Table 3:** Communication aspects including relationship, referrals and communication methods

Relationship and referrals	Physicians (*n* = 269)	Osteopaths (*n* = 297)
	Frequency (%)	Frequency (%)
Professional relationship (yes)	96 (35.6)	122 (41.1)
Referrals	Typical month	Last 6 months
0	104 (39.1)	107 (36.0)
1	55 (20.7)	45 (15.2)
2–3	42 (15.8)	79 (26.6)
4–5	13 (4.9)	33 (11.1)
More than 5	10 (3.8)	33 (11.1)
Never under any circumstances	42 (15.8)	n/a
New pediatric patients in osteopathic clinics referred by physicians over a two week period	n/a	269/1293 (20.8)
Written referral	96 (35.7)	96 (32.3)
Preferred primary communication method	Physicians (*n* = 107)	Osteopaths (*n* = 126)
	Frequency (%)	Frequency (%)
Letter	38 (35.5)	57 (45.2)
Verbal to patient	39 (36.4)	48 (38.1)
Phone	19 (17.8)	8 (6.3)
Email	5 (4.7)	7 (5.6)
In person	3 (2.8)	4 (3.2)
Fax	3 (2.8)	2 (1.6)
Importance of having the other professional among collaborators	Physicians (*n* = 259)	Osteopaths (*n* = 291)
	Frequency (%)	Frequency (%)
Not important	38 (14.7)	8 (2.8)
Slightly important	77 (29.7)	39 (13.4)
Quite important	113 (43.6)	163 (56.0)
Very important	31 (12.0)	81 (27.8)

Sample sizes varied due to non-responses

### Influence of regulation

The majority of physicians (72%) and osteopaths (62%) said they would be moderately or greatly influenced by the upcoming government regulation of osteopathy and the creation of university-based osteopathic programs in Quebec. Eighteen percent of physicians and 22% of osteopaths would not be very influenced and respectively 10% and 16% said they would not be influenced at all by regulation, either because they already collaborated or because they had no interest in physician-osteopath collaboration (comments on the survey).

### Factors associated with osteopathic referrals by physicians

Variables statistically associated with osteopathic referrals by physicians at the bivariate level were: gender (female), profession (family physician), general knowledge about practice parameters, direct sources of information about osteopathy, belief in the active role of osteopathy for pediatric conditions, community practice, personal consultation of an osteopath, and having a professional relationship. These last four variables remained in the final stepwise backward logistic regression model (Table 4).

**Table 4:** Factors associated with osteopathic referrals by physicians

	OR (95% CI)	*P* value
Gender (female)	1.97 (0.96, 4.02)	0.064
Direct source of information	1.93 (0.98, 3.81)	0.058
Belief in active role for pediatric conditions	1.22 (1.01, 1.47)	0.042
Community practice	1.89 (1.03, 3.47)	0.040
Personal consultation	2.58 (1.35, 4.93)	0.004
Professional relationship	4.10 (2.12, 7.95)	<0.001

Stepwise backward logistic regression

### Factors associated with knowledge about osteopathic general practice parameters

In the final linear regression model F (7247) = 9.117 (*p* < 0.001), 20.5% of the variance in the number of correct answers (knowledge) concerning general osteopathic practice parameters was explained by having a direct source of information about osteopathy (osteopath or other health professional; *p* = 0.009) and the number of positive answers for belief in the appropriateness of osteopathy for pediatric conditions (*p* < 0.001) while considering all the other bivariate associated variables in the model (professional relationship, personal consultation with an osteopath, osteopathic referrals, importance of having an osteopath among collaborators (quite or very important versus not important), and gender).

## Discussion

### Implication of the findings

The main aim of this study was to describe the current situation in Quebec respecting interprofessional collaboration between physicians and non-physician osteopaths working with pediatric patients. We found that some collaboration already exists, including referrals and professional relationships between these practitioners, but other factors might improve this collaboration. Having a professional relationship or having personally used osteopathic services were the factors most strongly associated with osteopathic referrals. It is known that consulting a CAM practitioner or having used CAM personally influences CAM referrals for the pediatric population [22, 23]. Indeed, as noted by Mior and collaborators (2010) in the early stages of implementing collaborative care between conventional and CAM practitioners, informal social meetings or events could foster the personal relationships that help to develop mutual respect and trust and break down barriers to communication.

The majority of the survey respondents acknowledged the importance of communication, including exchanging information about common patients. The preferred communication methods were verbal via the patients or in writing for practitioners. In a previous study, physicians who recommended that parents of young patients consult with a CAM practitioner reported that sustained two-way communication with CAM practitioners was rare [24]. In the present study, one third of respondents provided parents with a written referral, regardless of profession. In their cross-sectional study on communication regarding pediatric patients, Ben-Arye and collaborators (2007) found that, in addition to communicating clinical information and increasing the willingness to respond to the initial referral, the exchange of referral letters positively impacted the development of collaboration between physicians and CAM practitioners [25]. Letters about common patients should include conventional/CAM diagnoses using jargon-free terminology, possible conventional/CAM treatment interactions, and treatment plan and goals [26]. Since disclosure and overall discussion about CAM use during physician/patient encounters is reportedly limited [5, 27], especially for the pediatric population, verbal exchanges via the patient may not guarantee efficient communication between practitioners compared to exchanging letters.

Communication is also an important catalyst for other determinants of collaboration, such as sharing and developing mutual trust and respect [14]. Mutual respect implies knowledge and recognition of the complementarity of other professionals’ contributions [12]. Pediatricians who have a high level of knowledge about CAM are more likely to recommend CAM and discuss CAM with parents [23]. In this study, half the physicians asked an osteopath directly for information while about 20% asked other professionals such as colleagues about the role of osteopathy for pediatric patients. Having such direct sources of information and believing osteopathy to be appropriate for certain pediatric conditions are associated with physicians’ greater knowledge of general osteopathic practice parameters. More knowledge about practice parameters may lead to safer, appropriate two-way referrals. However, very few physicians said they had access to continuing education sessions about osteopathy, suggesting that formal education and information transmission concerning osteopathy are rare and could be improved.

Less than half the physicians knew that non-physician osteopaths are not yet regulated in Quebec, suggesting some confusion about the current status of osteopathic practice. However, the majority of respondents, both physicians and osteopaths, expected to be positively influenced by professional regulation and osteopathic university-based education. IPC between conventional and CAM practitioners is known to be affected by systemic determinants such as regulation [16]. For example, practice conditions governed by Colleges of Physicians and Surgeons were found to be a significant barrier to IPC [15, 28] and, even when referral to unregulated CAM is allowed, physicians must consider patients’ needs in light of medical, legal and ethical issues [29]. A positive view of the upcoming regulation and the creation of osteopathic university-based programs in Quebec might improve interactions between practitioners and increase interprofessional education for future practitioners. More interactions during the training phase might in turn diminish current power disparities observed in practice [30].

### Study limitations

This study has some limitations. First, the low response rate, especially for physicians, may limit the generalizability of the results. Although the response rate is typical for this type of survey [3], lack of time, low perceived relevance of the study topic or receiving more surveys a week might explain the physicians’ low response rate [31] compared to the osteopaths. Nearly half the physicians had personally used osteopathic services, which may suggest that those respondents already had a positive attitude toward osteopathy. Since the respondents appeared to be open to and in favour of collaboration, the results likely underestimate the challenges facing collaboration between physicians and osteopaths. To deepen our understanding of IPC between these practitioners, some of the results of this survey are being further explored with qualitative data from purposeful sampling of physicians and osteopaths (phase 2 of the study).

## Conclusions

This study examined the current IPC situation between physicians and non-physician osteopaths working with pediatric patients. Findings suggest that some collaboration already exists, including mutual referrals, but optimizing this collaboration still poses some challenges. Given the pending regulation of the osteopathic profession in Quebec and the need to promote the development of a healthcare delivery model that fosters safe patient-centred care, efforts must be made to reinforce communication skills and opportunities, provide physicians with easily accessible information about osteopathy, and ensure that regulatory bodies establish and maintain relationships after regulation.

## Abbreviations

CAM: Complementary and alternative medicine
IPC: Interprofessional collaboration
